# Megakaryocyte phenotyping in response to SARS-CoV-2 variants

**DOI:** 10.1080/09537104.2025.2532459

**Published:** 2025-07-23

**Authors:** Marcin A Sowa, Michael Tuen, Florencia Schlamp, Yuhe Xia, Marie I Samanovic, Mark J Mulligan, Tessa J Barrett

**Affiliations:** a Leon H. Charney Division of Cardiology, Department of Medicine, Cardiovascular Research Center, NYU Grossman School of Medicine, New York, NY, USA;; b NYU Langone Vaccine Center, and Division of Infectious Diseases and Immunology, Department of Medicine, NYU Grossman School of Medicine, New York, NY, USA

**Keywords:** COVID-19, CXCL8, megakaryocytes, platelets, thrombosis

## Abstract

SARS-CoV-2 infection is associated with platelet hyperreactivity and increased rates of arterial and venous thrombosis. SARS-CoV-2 mutations have resulted in several variants with differences in transmissibility, infectivity, and patient outcomes. This study investigates the effects of the ancestral strain of SARS-CoV-2 (WA1) and two variants of concern, Delta and Omicron, on the human megakaryocyte (MK) phenotype and transcriptome. Human CD34^+^-derived MKs were incubated with WA1, Delta or Omicron SARS-CoV-2 variants for 24 hours. MK activation markers were measured under resting and thrombin-stimulated conditions. RNA-seq and cytokine release in response to the viruses were assessed. Plasma cytokines were measured in hospitalized COVID-19 patients. Treatment of MKs with WA1, Delta or Omicron variants of SARS-CoV-2 resulted in similar increases in classical activation markers. However, SARS-CoV-2 variants mediated distinct transcriptomic changes. Across variants, 60 genes overlapped, including *CXCL8*. Consistent with transcriptomic changes, SARS-CoV-2-incubated MKs secreted significantly elevated levels of IL-8. Among hospitalized COVID-19 patients, plasma IL-8 levels were highest in COVID-19 patients who subsequently experienced thrombotic events or died. In conclusion, WA1, Delta, and Omicron similarly induce classical MK activation responses while mediating distinct transcriptomic changes. Increased IL-8 levels may serve as a biomarker to inform platelet hyperreactivity and thrombotic events associated with COVID-19.

## PLAIN LANGUAGE SUMMARY

**What is the context?:**

SARS-CoV-2 infection is associated with platelet hyperreactivity and increased rates of arterial and venous thrombosis. SARS-CoV-2 mutations have resulted in several variants with differences in transmissibility, infectivity, and patient outcomes. However, the specific transcriptomic and functional changes in megakaryocytes and platelets in response to infection by different SARS-CoV-2 variants remain largely unexplored.

**What is new?:**

This study investigates how different SARS-CoV-2 variants affect the function and transcriptome of platelet precursors – megakaryocytes. Exposure of megakaryocytes to various SARS-CoV-2 variants resulted in similar activation but distinct transcriptomic changes. SARS-CoV-2 increased IL-8 levels in megakaryocytes, and elevated IL-8 levels were also observed in plasma from COVID-19 patients. Notably, plasma IL-8 levels were highest in COVID-19 patients who subsequently experienced a thrombotic event or died.

**What is the impact?:**

Elevated IL-8 levels in MKs, independent of SARS-CoV-2 variant type, and in the plasma of COVID-19 patients suggest that IL-8 may serve as a biomarker for platelet hyperreactivity and thrombotic events associated with COVID-19.

## Introduction

Severe acute respiratory syndrome coronavirus 2 (SARS-CoV-2), the virus responsible for COVID-19, has caused a global pandemic, leading to significant morbidity and mortality worldwide. Since its emergence, SARS-CoV-2 has evolved into several variants, exhibiting differences in transmissibility, virulence, and immune escape capabilities.^1,2^ Infection and subsequent activation of platelets by SARS-CoV-2 is hypothesized to contribute to COVID-19-associated thrombosis.^3,4^ High rates of arterial and venous thrombosis are frequently seen in SARS-CoV-2 infection, significantly contributing to the morbidity and mortality associated with COVID-19.^5–8^

Current knowledge indicates that SARS-CoV-2 can induce significant platelet transcriptomic changes and cytokine release, leading to altered platelet phenotypes characterized by hyperreactivity.^9,10^ Furthermore, platelet precursors, megakaryocytes (MKs), have distinct phenotypic changes following SARS-CoV-2 infection that are associated with mortality and multiorgan injury.^11–13^ Beyond their traditional roles in hemostasis, both platelets and MKs are increasingly recognized as immune cells capable of sensing pathogens, releasing cytokines, and interacting with immune cells.^14–16^ This immunological functionality positions them as key players in the host response to viral infections, including SARS-CoV-2. However, there are limited studies on the effects of different SARS-CoV-2 variants on platelets and MKs. COVID-19 infection, particularly during the Delta variant peak, was associated with increased rates of thrombotic complications, highlighting the importance of vaccination and new anticoagulation strategies to mitigate risk.^17^ Additionally, the Omicron variant, despite generally causing less severe clinical signs compared to earlier variants, also mediates platelet activation and desensitization, similar to the Delta variant.^18^ Although these findings are significant, the specific transcriptomic and functional changes in MKs and platelets in response to infection by different SARS-CoV-2 variants remain largely unexplored.

This study aimed to investigate the effects of SARS-CoV-2 variants on MK function and transcriptome, explore their association with inflammatory biomarkers in COVID-19 patients, and highlight potential indicators for COVID-19-associated thrombotic complications. Although MKs do not directly participate in thrombus formation, they play a critical upstream role in determining platelet phenotypes. The CD34^+^-derived MK model used here has been widely applied as a surrogate to study how environmental stimuli, including viral infections and antiplatelet therapies, influence platelet programming.^19–22^

## Materials and methods

### Generation of virus stocks

Virus stocks were produced and purified using a 25% sucrose cushion, followed by resuspension in PBS; additional details are available in the Supplementary Materials.

### Megakaryocyte exposure to SARS-CoV-2

CD34^+^ cells collected before 2020 from four COVID-19-unvaccinated male donors (aged 30–42 years) were obtained from Fred Hutch Cancer Center (Seattle, WA). Cells were cultured in the presence of thrombopoietin to induce MK differentiation and maturation, as previously described.^20^ On day 11, cells were exposed to SARS-CoV-2 isolate WA1/2020, SARS-CoV-2 Delta, SARS-CoV-2 Omicron BA.1 at a multiplicity of infection (MOI) of 5 or vehicle control (PBS) for 24 hours. Following co-incubation, MKs were centrifuged, supernatants collected, and the cells were prepared for flow cytometry analysis or lysed to isolate RNA for RNA-seq. All SARS-CoV-2-related assays were performed in the CDC/USDA-approved Biosafety Level 3 facility of NYU Grossman School of Medicine, following its Biosafety Manual and Standard Operating Procedures.

### Megakaryocyte activation

5 × 10^4^ cells per condition resuspended in Tyrode’s buffer were plated on a 96-well plate, incubated with an antibody mix prepared in Tyrode’s buffer containing PAC-1-FITC (BD Biosciences), P-selectin-PE (BD Biosciences), CD63- Alexa Fluor^®^ 647 (BD Biosciences), CD61-PeCy7 (BioLegend) and activated with 0.2 or 0.5 U/mL thrombin (HemoIL^®^), 2 μg/mL convulxin (Santa Cruz Biotechnology) or vehicle control for 15 minutes at 37°C. Cells were fixed with 8% paraformaldehyde and analyzed using MACS Quant 16 (Miltenyi Biotec). The flow cytometry data were analyzed using FlowJo^™^ v10.8.1 Software (BD Life Sciences). Representative flow cytometry plots and gating strategies are provided in Fig. S1.

### Megakaryocyte RNA-seq

MK RNA sequencing and data processing were performed as we have previously described.^20^ Briefly, RNA-seq data from MKs were analyzed in R (v.4.0.2) with the package DESeq2 (v.1.24.0) using a linear model. Statistical significance was calculated using the Wald test. Genes with a base mean < 30 were excluded from further analysis. Pathway enrichment analysis was performed using Ingenuity Pathway Analysis (IPA) software (QIAGEN). Raw and processed RNA-seq data are available through the Gene Expression Omnibus GSE277576.

### Human cohort

A cohort of 291 hospitalized patients with COVID-19 and 21 patients without COVID-19 has been previously described.^9^ The study was approved by the NYU Grossman School of Medicine Institutional Review Board (IRB) and performed with a waiver of informed consent.

### Cytokine measures

IL-8 was measured in the supernatant of MKs using LEGENDplex according to the manufacturer’s instructions (BioLegend). In human plasma, IL-8, CD40L, CD62P, PSGL1 and IL-6 were measured using LEGENDplex (BioLegend), while TXB_2_ was measured using ELISA (Cayman Chemical) according to the respective manufacturers instructions.

### Statistical analyses

Data are presented as mean ± standard deviation (SD). One-way ANOVA was performed when three or more groups were compared for one variable, followed by Dunnett’s post-hoc test. The transcriptomic analysis utilized nominal *P-values* assessed by DESeq2. The association between groups was determined using Spearman’s correlation coefficient (*R*). A two-sided unpaired parametric Student’s *t*-test was used for within-group comparisons. The Mann-Whitney U-test was used for the analysis of continuous variables (demographics). Differences between categorical variables were calculated using chi-square or Fisher’s exact test, as appropriate. Logistic regression was used to assess the association between IL-8 and outcomes. Results are presented as odds ratios (ORs) along with their 95% confidence intervals (CIs). *P-values* < 0.05 were considered statistically significant. Statistical analyses were performed with GraphPad Prism 10.1.1 (GraphPad Software) and R (v.3.5.2).

## Results

**Figure 1.**
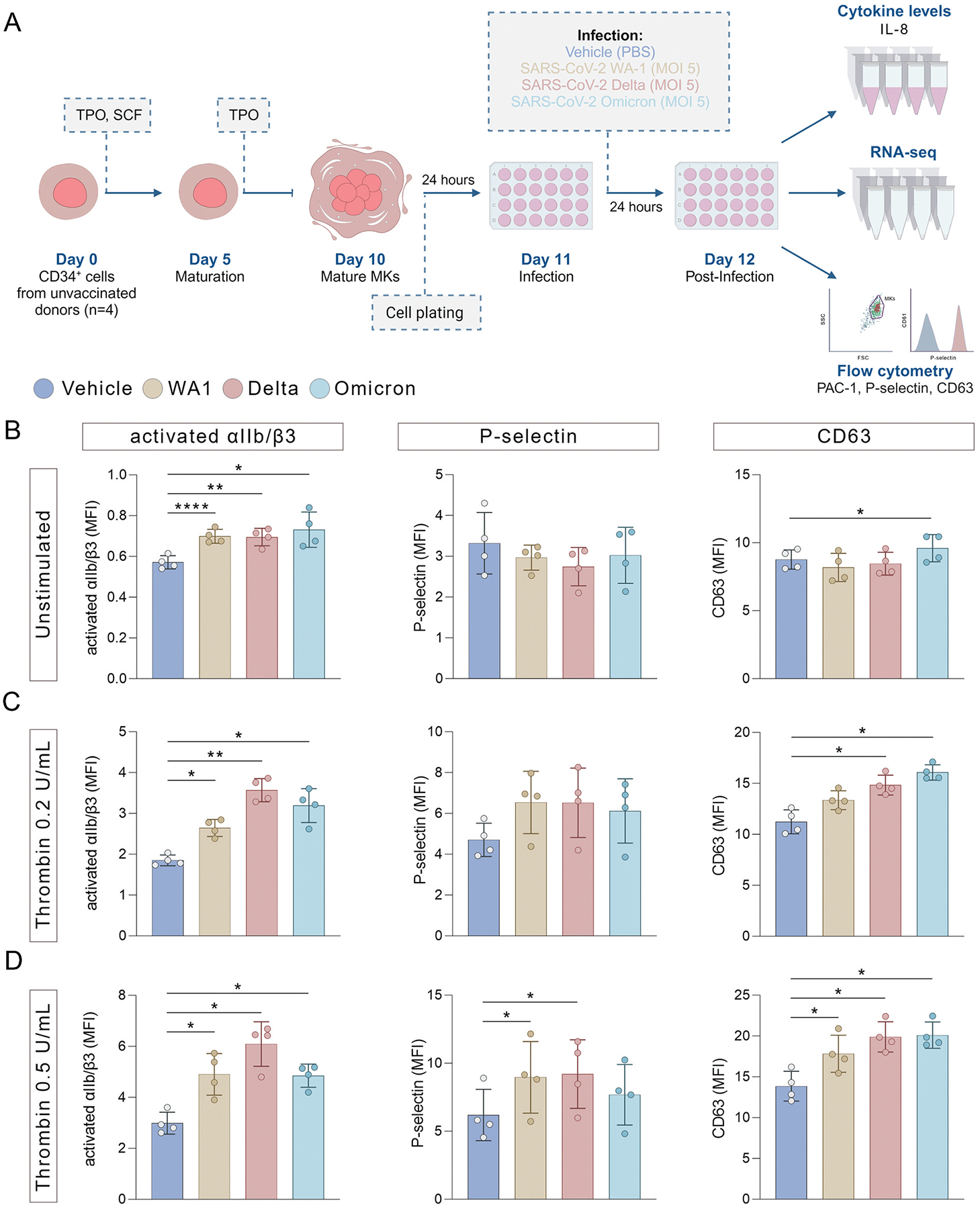
SARS-CoV-2 variants potentiate classical megakaryocyte activation responses to a similar degree. (A) CD34^+^ cells from four COVID-19 unvaccinated donors were differentiated into MKs for 5 days in the presence of human thrombopoietin (TPO) and human stem cell factor (SCF), and then until day 10 in the presence of TPO only. On day 11, the cells were co-incubated with WA1, Delta or Omicron SARS-CoV-2 isolates or vehicle control for 24 hours. After 24 hours, supernatants were collected for cytokine level quantification, and cells were collected for either RNA-seq or flow cytometry analysis of activation markers. (B-D) On day 12, MKs were unstimulated (B) or treated for 15 minutes with 0.2 U/mL or 0.5 U/mL thrombin (C and D, respectively), stained for activated αIIb/β3 (PAC-1), P-selectin and CD63 and analyzed by multicolor flow cytometry. The geometric mean fluorescence intensity (MFI) of PAC-1 binding, P-selectin expression and CD63 expression in response to an agonist was recorded as a measure of reactivity. Data are shown as mean ± SD and represent four independent unvaccinated donors. *P-values* were calculated by one-way ANOVA with Dunnett’s post-test (*P < .05, **P < .01, ***P < .001, ****P < .0001).

To investigate the functional and transcriptomic consequences of MK SARS-CoV-2 co-incubation, mature CD34^+^-derived MKs (Figure S2) were incubated with purified SARS-CoV-2 ancestral strain WA1, Delta or Omicron for 24 hours (Figure 1A). MK integrin αIIb/β3 activation and degranulation were measured at baseline and following stimulation with 0.2 and 0.5 U/mL thrombin or 2 μg/mL convulxin. In unstimulated MKs, SARS-CoV-2 exposure with the ancestral isolate and all variants compared to vehicle control significantly increased activated integrin αIIb/β3 expression while P-selectin expression remained unchanged (Figure 1B). A small but significant increase in MK CD63 expression was found with the Omicron variant exposure (Figure 1B). Similarly, following stimulation with low-dose thrombin (0.2 U/mL), activated integrin αIIb/β3 expression increased across all exposures with ancestral isolate and variants, P-selectin levels remained unchanged, while CD63 expression increased following contact with both Delta and Omicron variants (Figure 1C). When stimulated with 0.5 U/mL thrombin, MK activated αIIb/β3 expression increased with ancestral isolate and variants, P-selectin expression increased with WA1 and Delta variant co-incubations, and CD63 expression increased for all isolates (Figure 1D). However, stimulation with 2 μg/mL convulxin did not show differences between SARS-CoV-2 variants and vehicle control, increasing activated αIIb/β3, P-selectin, and CD63 to a similar degree (Figure S3).

**Figure 2.**
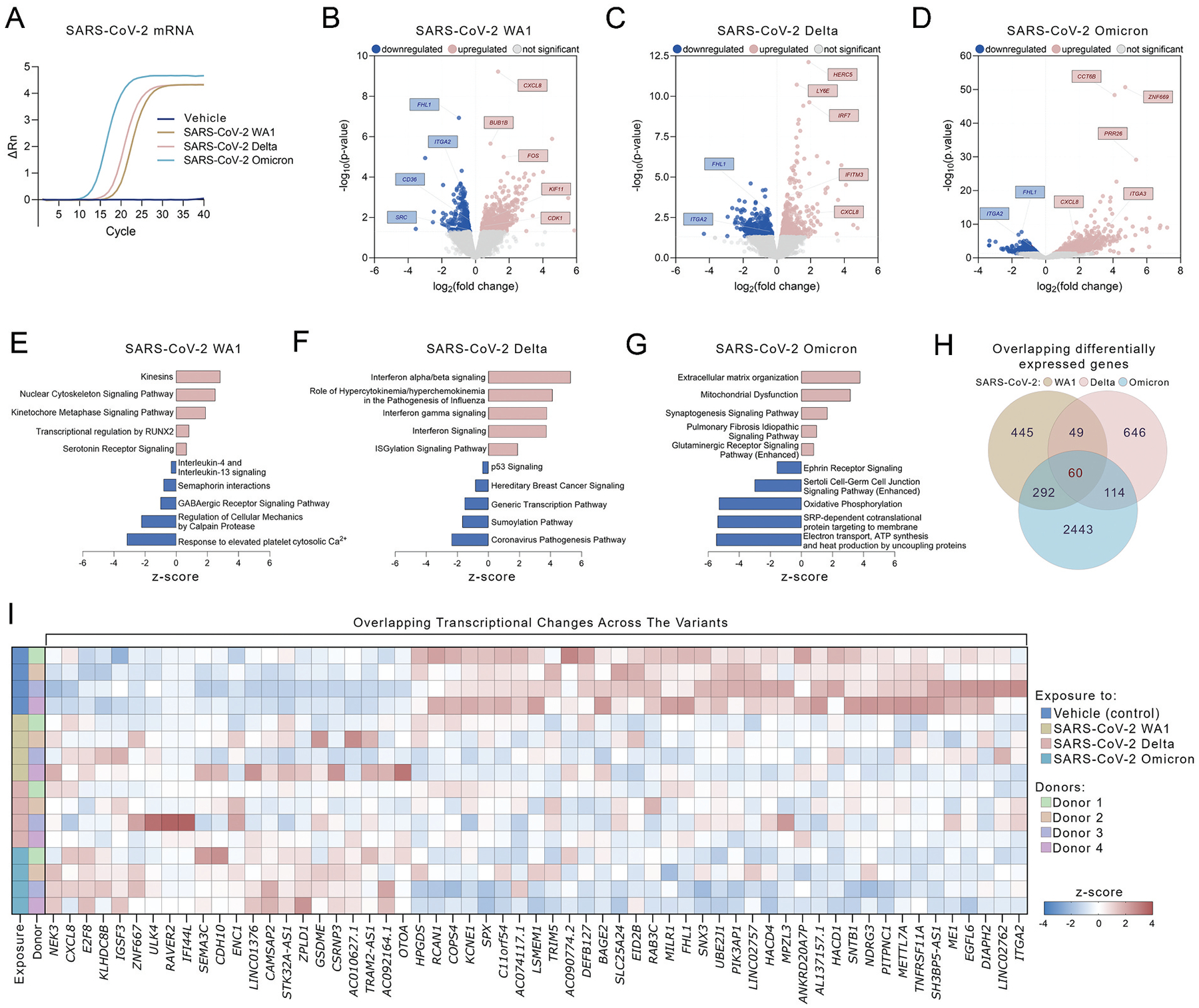
SARS-CoV-2 variants have distinct effects on the megakaryocyte transcriptome. (A) Amplification plots of SARS-CoV-2-specific real-time polymerase chain reaction (RT-qPCR) of CD34^+^-derived MKs co-incubated with WA1, Delta or Omicron SARS-CoV-2 isolates or vehicle control (*n* = 4) for 24 hours. (B-D) Volcano plot of differentially expressed genes (DEGs) between vehicle control and SARS-CoV-2 WA1, Delta or Omicron (B, C and D, respectively). Colored dots are *p* < .05; red dots represent up-regulated and blue dots down-regulated genes. (E-G) Ingenuity Pathway Analysis (IPA) of DEGs identified upon co-incubation with SARS-CoV-2 WA1, Delta or Omicron (E, F and G, respectively). The top 5 up-regulated and top 5 down-regulated pathways are presented for each SARS-CoV-2 variant (*p* < .05). (H) Venn diagram depicting shared DEGs between SARS-CoV-2 isolates. (I) Heatmap of overlapping DEGs between SARS-CoV-2 variants. Z-scores were computed gene-by-gene by subtracting the mean of normalized counts and dividing by the standard deviation.

RNA-seq was performed to investigate the impact of SARS-CoV-2 variants on altering the MK transcriptome. SARS-CoV-2 mRNA was readily detected in MKs following co-incubation with all variants (Figure 2A). Compared to vehicle control-treated MKs, differentially expressed genes (DEGs) were identified upon contact with SARS-CoV-2 WA1 (*n* = 846), Delta (*n* = 869), and Omicron (*n* = 2909) (*p* < .05, Figure 2B–D).

Co-incubation with the WA1 resulted in upregulation of genes involved in cell division, RUNX2 signaling, and serotonin signaling. In contrast, genes associated with platelet function, cellular mechanics, and immune response were downregulated (Figure 2E). The Delta variant induced upregulation of pathways related to interferon signaling, and antiviral defense pathways, while pathways associated with sumoylation and transcription were downregulated (Figure 2F). Omicron exposure led to the upregulation of genes linked to cellular structure, mitochondrial dysfunction, and glutaminergic receptor signaling, and downregulation of cellular energy production and oxidative phosphorylation (Figure 2G).

Across the variants, 60 overlapping genes were identified (Figure 2H). Of these, 59 showed consistent expression patterns following virus treatment, with 22 genes upregulated and 37 downregulated (Figure 2I). Notably, the consistently upregulated, variant-independent genes included those involved in immune signaling (*CXCL8, IFI44L*), cell cycle regulation (*E2F8, KLHDC8B*), and apoptosis (*GSDME*), while downregulated genes were enriched for roles in metabolism (*ME1*), vesicle trafficking (*SNX3*), and immune modulation (*TRIM5, RCAN1*).

**Figure 3.**
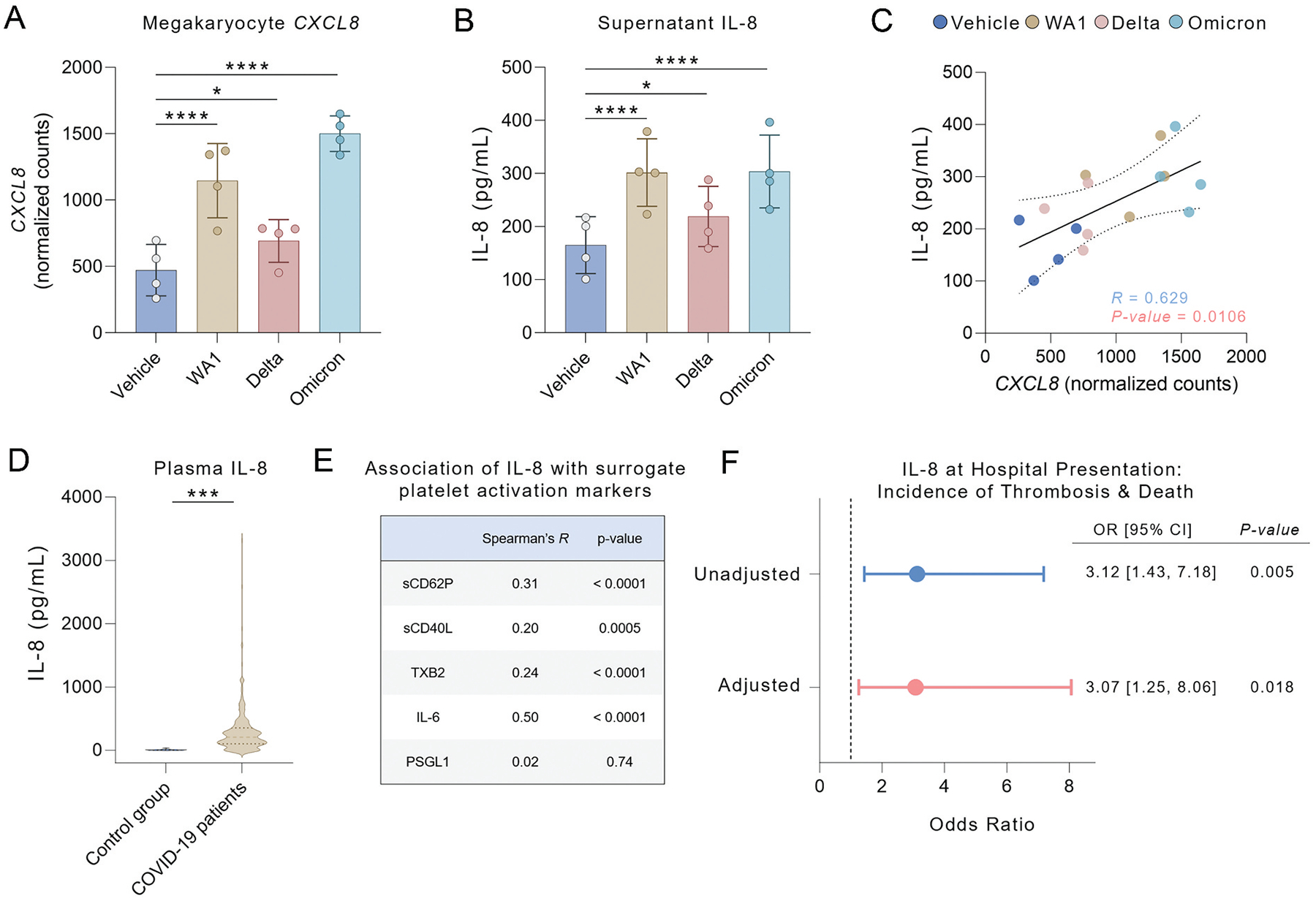
SARS-CoV-2 increases IL-8 release from megakaryocytes and in the plasma from COVID-19 patients. (A) Normalized expression counts of *CXCL8* of MKs infected with WA1, Delta or Omicron SARS-CoV-2 variants or vehicle control for 24 hours (*n* = 4). (B) Quantification of IL-8 release from MKs infected with WA1, Delta or Omicron SARS-CoV-2 isolates or vehicle control for 24 hours (*n* = 4). Data are shown as mean ± SD and represent four independent unvaccinated donors. *P-values* were calculated by one-way ANOVA with Dunnett’s posttest (*p < .05, **p < .01, ***p < .001, ****p < .0001). (C) Correlation between *CXCL8* normalized expression counts and IL-8 level in supernatant in MKs. The association between groups was determined using Spearman’s correlation coefficient (*R*). (D) Plasma level of IL-8 in the control group (*n* = 21) versus patients with COVID-19 (*n* = 291). Data are shown as mean ± SD. *P-values* were calculated using an unpaired parametric Student’s t-test (*p < .05, **p < .01, ***p < .001, ****p < .0001). (E) Correlation between plasma level of IL-8 and plasma levels of interleukin 6 (IL-6), soluble CD40 ligand (sCD40L), soluble P-selectin, thromboxane B_2_ (TXB_2_), P-selectin glycoprotein ligand-1 (PSGL-1). Plasma cytokine levels were measured in COVID-19 (*n* = 291) with a LEGENDplex assay or ELISA. The associations between IL-8 and inflammatory biomarkers were determined using Spearman’s correlation coefficient (*R*). (F) Odds ratio (OR) and adjusted OR with corresponding 95% confidence intervals (CIs) from logistic regression analysis for the outcome of thrombosis & death, based on admission plasma IL-8 levels in COVID-19 patients, comparing those in the 1st and 4th quartiles. Adjusted for age, sex, race/ethnicity, BMI, diabetes, COPD/asthma, and history of coronary artery disease or cancer.

CXCL8 is a key cytokine in COVID-19 pathogenesis, associated with adverse clinical outcomes and disease severity.^23,24^ Given that *CXCL8* was found to be upregulated across the SARS-CoV-2 ancestral strain and variants (Figure 3A), interleukin 8 (IL-8) levels were measured in the supernatant from SARS-CoV-2-exposed MKs. Relative to control-treated MKs, WA1, Delta, and Omicron viruses increased MK production of IL-8 (Figure 3B), with supernatant levels positively associated with MK *CXCL8* mRNA (*R* = 0.629, Figure 3C).

Consistent with COVID-19-associated systemic inflammation, we found that plasma IL-8 levels were significantly higher in COVID-19 patients (*n* = 291) compared to the control group (*n* = 21, Figure 3D). Additionally, plasma IL-8 levels positively correlated with the surrogate biomarkers of platelet activation CD40L, CD62P and TXB_2_ (Figure 3E). While IL-8 positively correlated with interleukin 6 (IL-6), no significant association was found between IL-8 and the monocyte activation marker P-selectin glycoprotein ligand-1 (PSGL-1).

Among the COVID-19 patients, IL-8 levels were highest in COVID-19 patients who subsequently experienced a thrombotic event or died (median [IQR]: 246.00 pg/mL [139.25, 432.75] versus 179.00 pg/mL [80.00, 327.00], *p* = .006, Table 1). Patients with plasma IL-8 levels in the highest quartile had a greater than three-fold odds of thrombosis or death (odds ratio [OR] = 3.12 [1.43 to 7.18], *p* = .005, Figure 3F), which remained significant following adjustment for age, sex, race/ethnicity, BMI, diabetes, COPD/asthma, and history of coronary artery disease or cancer (adjusted OR [adjOR] = 3.07 [1.25 to 8.06], *p* = .018, Figure 3F). These data indicate that IL-8 may serve as a biomarker to inform platelet hyperreactivity and thrombotic risk in hospitalized COVID-19 patients.

## Discussion

Inflammation and thrombosis are both clinical manifestations of SARS-CoV-2 infection. Platelets can interact with viruses and participate in both inflammation and thrombosis, and several studies have also highlighted that MKs are important contributors to COVID-19-associated platelet dysfunction and hyperinflammation.^11,25,26^ Our study aligns with the reports of MK and platelet activation and reactivity in COVID-19 patients.

This study demonstrates increased MK cytokine production and sensitivity to protease-activated receptor (PAR) agonists following exposure to SARS-CoV-2, which supports studies demonstrating platelet hyperreactivity in COVID-19 patients and increased thrombotic risk.^27–29^ In the absence of thrombin stimulation, SARS-CoV-2 exposure induced only modest activation of MKs. This observation may suggest that SARS-CoV-2 does not fully activate MKs on its own but instead primes them for enhanced responsiveness to coagulation signals. Such priming could amplify the effects of thrombin and other coagulation factors *in vivo*, contributing to the hypercoagulable state observed in COVID-19.^30^ Further studies are needed to elucidate the molecular mechanisms underlying this potential priming effect and its relevance to disease progression. Moreover, the more robust activation of integrin αIIb/β3 compared to P-selectin observed in our study may reflect the differential regulation of these markers by distinct intracellular signaling pathways. Transcriptomic analysis revealed that SARS-CoV-2 exposure, particularly with the Delta variant, upregulated genes involved in cytoskeletal remodeling and integrin signaling, which are central to the inside-out activation of αIIb/β3. These findings suggest that SARS-CoV-2 may selectively prime MKs for integrin activation without broadly triggering granule release. In contrast to previous studies that have shown differential platelet activation responses following exposure to Delta and Omicron variants, we found MK activation responses to be similar across variants.^18,31^ Inherent differences between MKs and platelets, such as their environments or timing of viral infection degree, could lead to the phenomenon known as “exhausted platelets,” which subsequently triggers the shedding process.^32^ Moreover, although virus stocks were purified, we cannot exclude the possibility that factors derived from either the virus or infected host cells contributed to megakaryocyte activation, as current purification methods do not fully eliminate host-derived components. This includes extracellular vesicles and membrane-associated proteins such as tissue factor, which have been shown to play a role in coagulation and immune activation.^33–35^

While previous platelet transcriptomic studies in COVID-19 patients did not specify the type of variant,^9,10^ a study involving mice expressing human ACE2 analyzed transcriptomic changes in MKs upon infection with Delta variant and found that processes such as histone modifications, MK differentiation and NETosis were affected in the MKs of COVID-19-infected mice.^13^ Notably, our findings suggest that the Delta variant was the most proinflammatory, characterized by significant upregulation of interferon and ISGylation signaling pathways and hypercytokinemia. Moreover, our transcriptomic analysis of MKs infected with the Delta variant identified a significant upregulation of interferon-induced transmembrane protein 3 (*IFITM3*), known to inhibit viral replication. This finding aligns with the observed upregulation of IFITM3 in platelets from patients infected with the Delta variant^18^ and platelets and MKs of COVID-19 patients.^10,11^ In contrast, other variants exhibited distinct transcriptomic profiles, with WA1 and Omicron showing unique patterns of gene regulation that reflect their differing impacts on cellular processes.

*CXCL8* was found upregulated in MKs across all SARS-CoV-2 variants. Previously, IL-8 has been identified as a key cytokine in COVID-19, serving as a strong stratifier for clinical outcomes and a biomarker for predicting disease severity and prognosis^23,24^ and upregulated in SARS-CoV-2 positive MKs,^11^ suggesting that IL-8 plays a crucial role in the inflammatory and thrombotic processes associated with COVID-19. The consistent upregulation of *CXCL8* across the SARS-CoV-2 ancestral strain and variants, along with elevated IL-8 release from MKs exposed to SARS-CoV-2 variants and in COVID-19 patient plasma, underscores its potential as a biomarker for disease severity. However, although elevated IL-8 levels are associated with an increased risk of thrombosis and death in COVID-19 patients, it is important to note that IL-8 can also be released by various other cell types, including monocytes, neutrophils, endothelial cells, epithelial cells and fibroblasts.^36^

The selective increase in IL-8 following SARS-CoV-2 exposure suggests that the virus may promote the targeted release of specific alpha granule contents rather than inducing broad degranulation. IL-8, stored in platelet alpha granules, was upregulated at both transcript and protein levels in MKs, indicating potential transcriptional priming or selective trafficking. In contrast, P-selectin expression remained largely unchanged, further supporting the idea that SARS-CoV-2 may not induce widespread granule release but instead favors the secretion of specific inflammatory mediators. However, since we did not assess other granule constituents, the extent and specificity of this selective release remain unclear.

Of note, other studies have found that IL-8, IL-6, CD40L, P-selectin, and TXB_2_ are significantly increased in the plasma of COVID-19 patients, further supporting their roles in the disease’s inflammatory and thrombotic pathways.^10,11,18,27,31,37,38^ While the following markers are not exclusively platelet-specific, they provide valuable insights into the overall inflammatory and thrombotic state during SARS-CoV-2 infection.

This study has several limitations. First, we do not provide definitive proof of infection, as detection of MK SARS-CoV-2 mRNA may originate from non-replicative viral particles and may miss the dynamic changes over time and stages of viral infection. Additional assessments, such as plaque assays or viral protein production, are necessary to confirm active infection. Second, the *in vitro* model may not fully replicate the complex interactions and environment found in living organisms. Third, the limited sample size (*n* = 4) may reduce the statistical power and generalizability of the findings. Fourth, given the uncertain significance of IL-8 release from MKs and the possibility that it may be related to the biological function of MKs rather than directly associated with platelet hyperreactivity, the contribution of IL-8 needs to be further validated in independent cohorts to substantiate its role as a biomarker for platelet hyperreactivity and thrombotic events. Last, we did not assess MK ploidy or stratify responses by maturation state, which limits conclusions about ploidy-specific susceptibility. However, MKs are known to exhibit transcriptional diversity, and some subsets may be more responsive to inflammatory cues or viral exposure. These immune-responsive MKs could differ in their susceptibility to infection and may influence both their maturation and the characteristics of the platelets they produce.

In conclusion, we found similar upregulation of MK activation markers and distinct transcriptional changes following co-incubation with ancestral WA1 SARS-CoV-2 and its variants, Delta and Omicron. Moreover, we observed increased release of IL-8 from MKs following virus exposure, consistent with increased plasma IL-8 levels in COVID-19 patients. Finally, we found a positive correlation between IL-8 release and surrogates of platelet hyperreactivity CD40L, P-selectin, TXB_2_, and IL-6. These findings highlight the significant impact of SARS-CoV-2 variants on MK function and suggest potential biomarkers for thrombotic complications in COVID-19 patients.

## Supplementary Material

Supp 1

Supplemental data for this article can be accessed online at https://doi.org/10.1080/09537104.2025.2532459.

## Figures and Tables

**Table 1.** Demographics of COVID-19 patients stratified by thrombosis or death.

	No Thrombosis or Death	Thrombosis or Death	*P*
**n**	205	86	
**Age**, median [IQR]	64.00 [50.00, 73.00]	69.00 [60.00, 78.00]	0.001
**Sex**, Female, n (%)	90 (43.9)	32 (37.2)	0.355
**Race**			0.313
White, n (%)	98 (47.8)	50 (58.1)	
African American, n (%)	30 (14.6)	12 (14)	
Hispanic, n (%)	4 (2.0)	2 (2.3)	
Asian, n (%)	28 (13.7)	5 (5.8)	
Other, n (%)	45 (22.0)	17 (19.8)	
**BMI**, median [IQR]	27.14 [23.63, 31.46]	27.85 [23.61, 33.28]	0.572
**Asthma**, n (%)	20 (9.8)	6 (7.0)	0.594
**Chronic obstructive pulmonary disease**, n (%)	7 (3.4)	8 (9.3)	0.075
**Coronary artery disease**, n (%)	25 (12.2)	12 (14.0)	0.827
**Current smoker**, n (%)	7 (3.4)	3 (3.5)	0.528
**Diabetes**, n (%)	52 (25.4)	24 (27.9)	0.761
**Hyperlipidemia**, n (%)	50 (24.4)	25 (29.1)	0.493
**Hypertension**, n (%)	98 (47.8)	50 (58.1)	0.139
**IL-8**, median [IQR]	179.00 [80.00, 327.00]	246.00 [139.25, 432.75]	0.006
