# Supplementary material for: Megakaryocyte phenotyping in response to SARS-CoV-2 variants
Source: Platelets. Author manuscript; Available in PMC 2025 Aug 13. (PMC12347718; doi:10.1080/09537104.2025.2532459)
Supplement: Supp 1 [file NIHMS2102324-supplement-Supp_1.docx]

**Supplementary materials**

*Sowa et al. Megakaryocyte Phenotyping in Response to SARS-CoV-2 Variants.*

**Supplementary Materials and Methods**

**Cell lines**

Vero E6-TMPRSS2-T2A-ACE2 cells (BEI #NR-54970) were cultured in DMEM (Corning 10-013-CV) containing 4 mM L-glutamine, 4500 mg per L glucose, 1 mM sodium pyruvate and 1500 mg per L sodium bicarbonate, supplemented with 10% fetal bovine serum and 10 μg per mL puromycin (Sigma) at 37°C with 5% CO2. Vero E6 (African Green Monkey Kidney; ATCC #CRL-1586) were cultured in Dulbecco’s Modified Eagle Medium (DMEM) (Gibco #11956126) supplemented with 10% heat-inactivated fetal bovine serum (FBS), 2 mM L-glutamine, and 1% of MEM Nonessential Amino Acid (NEAA) Solution (Thermo Fisher Scientific, #MT25025CI).

**Generation of Virus Stocks**

Virus stocks were obtained as follows, SARS-Related Coronavirus 2, Isolate USA-WA1/2020, BEI Resources, NR-52281; SARS-CoV-2 Delta variant (hCoV19/USA/PHC658/2021) and SARS-CoV-2 Omicron BA.1 (hCoV19/USA/GA-EHC-2811C/2021) were kindly provided by the Suthar Lab at Emory University. All SARSCoV-2 viruses were produced in Vero E6 cells and infected at a multiplicity of infection (MOI) of 0.01. Cells were harvested when 50% cytopathic effect (CPE) was observed. After harvesting, cellular debris was removed via centrifugation. Next, the virus was purified using a 25% sucrose cushion at 25,000 RPM for 4 hours and resuspended in 1-2 ml 1X PBS (Phosphate Buffered Saline) (Gibco #14190136). This produced purified SARS-CoV-2 stocks in a high concentration.

**SARS-CoV-2 titers**

Viral titers were determined by plaque assay. Briefly, viruses were diluted in 10-fold dilutions and added to a monolayer of Vero E6TMPRSS2-T2A-ACE2 cells and incubated for 1 hour at 37°C, on a shaker platform. Next, a 0.8% agarose solution in DMEM supplemented with 2%FBS was overlayed and incubated for 36-48 hours at 370C, 5% CO2. The cells were then fixed with a 10% formalin solution and agar plugs were removed. Cells were then stained with crystal violet and plaques were counted.

All SARS-CoV-2 infection assays were performed in the CDC/USDA-approved BSL3 facility of NYU Grossman School of Medicine, in accordance with its Biosafety Manual and Standard Operating Procedures.

**RNA isolation and RT-qPCR**

Total RNA was extracted using a Direct-zol RNA Purification Kit (Zymo Research). To quantify mRNA, cDNA was synthesized from 100 ng of RNA using the iScript cDNA Synthesis Kit (Bio-Rad) in a 10 μL reaction. The resulting cDNA was then diluted 1:9. Quantitative real-time PCR was conducted using a QuantStudio 3 (Thermo Fisher), the following primers targeting SARS-CoV-2 N protein: 5’-ATGCTGCAATCGTGCTACAA-3’ and 5’-GACTGCCGCCTCTGCTC-3’) and SYBR Green (Applied Biosystems). For each reaction, 2 μL of the diluted cDNA (equivalent to 2.2 ng) was used.

**Megakaryocyte Ploidy Assay**

0.5×10^6^ CD34^+^-derived MK on day 12 were centrifuged at 300g for 5 minutes, resuspended in FACS buffer (2% FBS in PBS) and stained for CD41-FITC (BD Pharmingen) and CD42d-APC (Invitrogen) on ice for 30 minutes. The cells were centrifuged at 300g for 5 minutes, resuspended in 70% ice-cold ethanol and incubated on ice for 30 minutes. Following another centrifugation at 300g for 5 minutes, the cells were resuspended in FACS buffer containing 50 µg/mL of propidium iodide (Sigma-Aldrich) and 50 µg/mL RNase A (Thermo Scientific) and incubated at room temperature for 20 minutes. The cells were then centrifuged at 300g for 5 minutes, washed once with FACS buffer, resuspended in FACS buffer and analyzed using MACS Quant 16 (Miltenyi Biotec). The ploidy distribution was analyzed using FlowJo™ v10.8.1 Software (BD Life Sciences).

**Phenotyping of megakaryocytic differentiation of CD34^+^ cells**

CD34^+^ hematopoietic stem cells were harvested at various time points of maturation (days 1, 5, 8, and 12) by centrifugation at 300g for 10 minutes. The cells were then resuspended in PBS and incubated with Human BD Fc Block (BD Biosciences) for 10 minutes at room temperature. Subsequently, the cells were stained with CD34-FITC (Invitrogen), CD42b-APC (BD Biosciences) and CD41a-PeCy7 (BD Biosciences) antibodies or IgG controls for 15 minutes at room temperature. Finally, the cells were fixed with 4% PFA and analyzed using the Miltenyi MACSQuant Analyzer 16 (Miltenyi Biotec).

**Supplementary Figures**


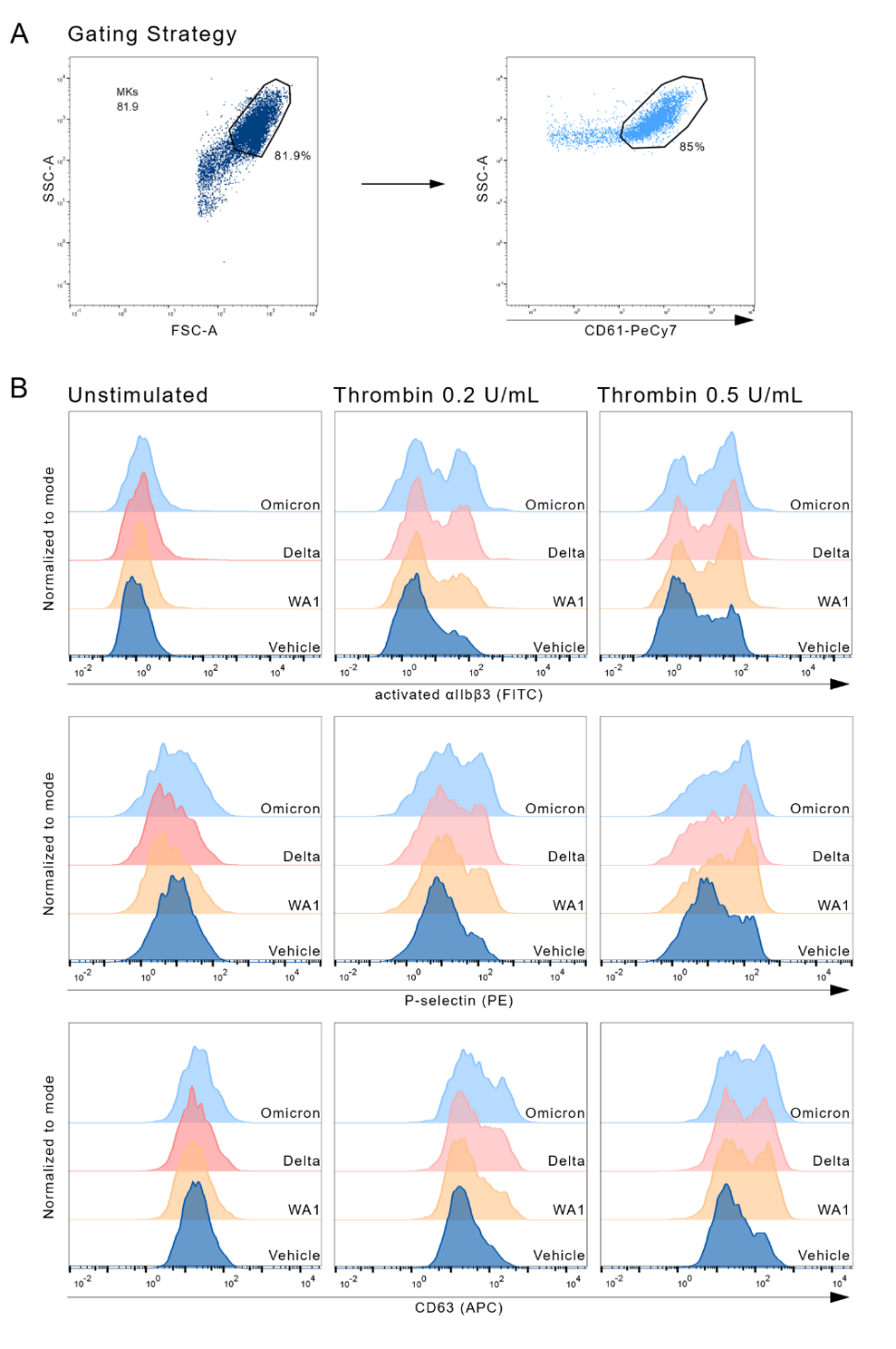


**Supplementary Figure 1. SARS-CoV-2 Variants Similarly Enhance Classical Activation Responses in Megakaryocytes.**

(A) Representative flow cytometry gating strategy: MKs were first gated based on size and granularity using forward scatter (FSC-A) and side scatter (SSC-A), followed by selection of CD61-PeCy7-positive cells to identify mature MKs. Expression of classical activation markers, activated αIIbβ3, P-selectin, and CD63, was then assessed within the CD61+ population. (B) Representative histogram plots showing expression levels of activated αIIbβ3, P-selectin, and CD63 in unstimulated MKs and those treated with 0.2 U/mL or 0.5 U/mL thrombin. Comparisons are shown across MKs treated with different SARS-CoV-2 variants (WA1, Delta, and Omicron) and vehicle control.


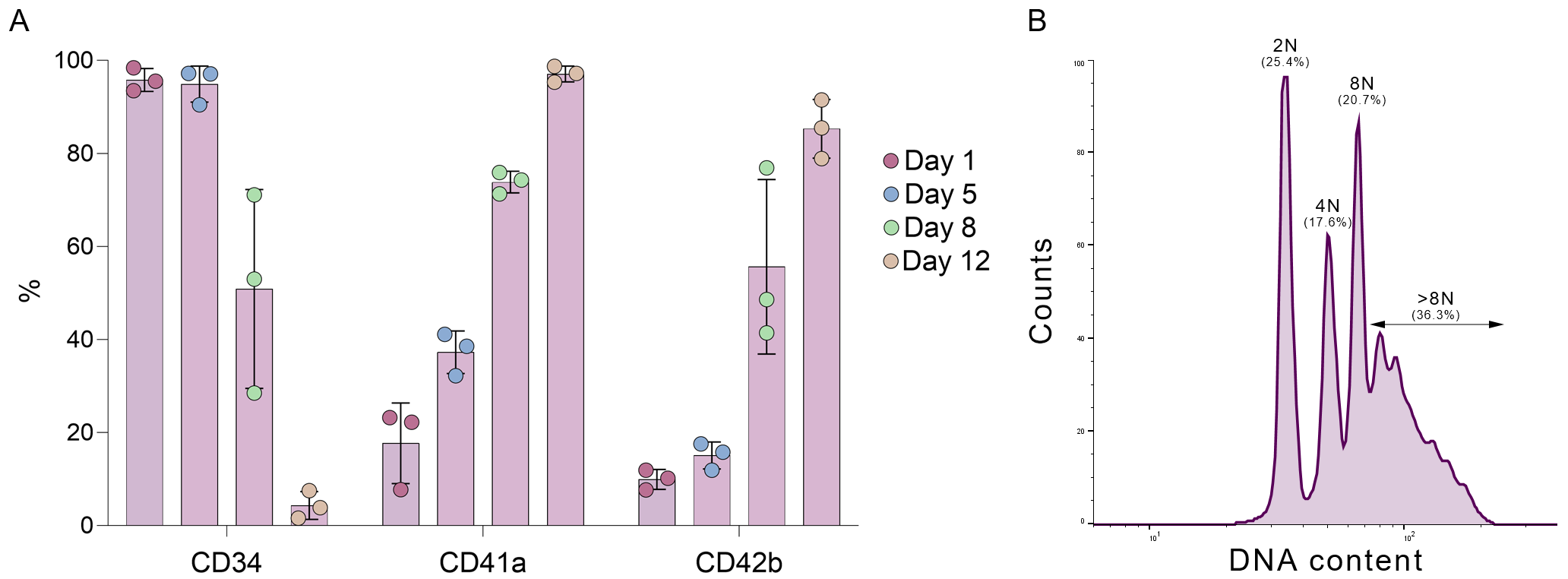


**Supplementary Figure 2. Megakaryocyte Ploidy and Megakaryocytic Differentiation of CD34^+^ Hematopoietic Stem Cells.**

CD34^+^ cells were differentiated in the presence of thrombopoietin and analyzed by flow cytometry to assess the ploidy and surface expression levels of megakaryocyte markers. (A) Percentage of cells expressing CD34, CD41a and CD42b over the differentiation time-points (n = 3 unique CD34^+^ donors). (B) Ploidy analysis of mature CD41^+^CD42d^+^ human megakaryocytes stained with propidium iodide to detect DNA content. Peaks representing 2N, 4N or 8N cell ploidy and a region representing >8N ploidy are indicated with the percentage of each ploidy level.


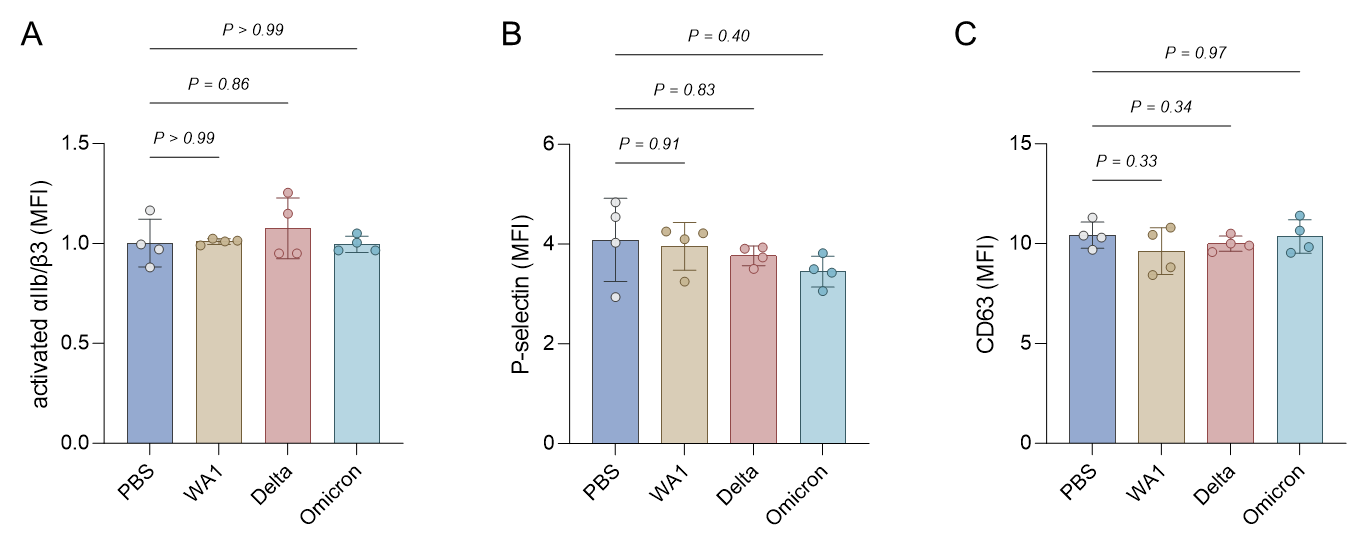


**Supplementary Figure 3. SARS-Cov-2 Variants Do Not Change Expression of Classical Megakaryocyte Activation Markers in Response to Convulxin.**

On day 11, MKs were co-incubated with WA1, Delta or Omicron SARS-CoV-2 isolates or vehicle control for 24 hours. After 24 hours, MKs were treated for 15 minutes with 2 µg/mL convulxin, stained for activated αIIb/β3 (PAC-1), P-selectin and CD63 and analyzed by multicolor flow cytometry. The geometric mean fluorescence intensity (MFI) of PAC-1 binding (A), P-selectin (B) and CD63 (C) in response to an agonist was recorded as a measure of reactivity. Data are shown as mean ± SD and represent four independent unvaccinated donors. P-values were calculated by one-way ANOVA with Dunnett’s post-test.
